# The sensitivity of acute flaccid paralysis surveillance - the case of South Sudan: retrospective secondary analysis of AFP surveillance data 2014-2019

**DOI:** 10.11604/pamj.supp.2022.42.1.33965

**Published:** 2022-06-11

**Authors:** Ayesheshem Ademe Tegegne, Sylvester Maleghemi, Atem Nathan Anyuon, Fikiru Abebe Zeleke, George Awzenio Legge, Melisachew Adane Ferede, Peter Daudi Manyanga, Victor Guma Paul, Nganda Moses Mutebi, Johnson Muluh Ticha, Ochan Taban David Kilo, Fabian Ndenzako, Mkanda Pascal, Olushayo Oluseun Olu

**Affiliations:** 1 World Health Organization Country Office, Juba, South Sudan,; 2 Federal Ministry of Health, Juba, South Sudan,; 3 World Health Organization Inter-Country Support Team for Eastern and Southern African Countries, Harare, Zimbabwe,; 4 World Health Organization African Region, Brazzaville, Congo

**Keywords:** Acute flaccid paralysis surveillance, South Sudan, poliovirus, surveillance sensitivity, surveillance performance

## Abstract

**Introduction:**

South Sudan has made quite impressive progress in interrupting wild poliovirus and maintaining a polio-free status since the last case was reported in 2009. South Sudan introduced different complementary strategies to enhance acute flaccid paralysis (AFP) surveillance. Hence, the objective of this study is to evaluate the sensitivity of the surveillance system using the WHO recommended surveillance standard and highlight the progress and challenges over the years.

**Methods:**

we conducted a retrospective, descriptive, quantitative study design and used the available secondary AFP surveillance database.

**Results:**

the overall non-polio AFP rate was 6.2/100,000 children under 15 years old in the study period. The stool adequacy was maintained well above the certification level of surveillance. The two main surveillance performance indicators were met at the national level throughout the study period. In contrast, only five out of ten states persistently attained and maintained the two main surveillance performance indicators throughout the study period, while in 2019 all states achieved except for Jonglei state. During the analysis period, no wild poliovirus was isolated except two circulating Vaccine Derived Poliovirus Type 2 (cVDPV2) cases in 2014 and one Immunodeficiency Vaccine Derived Poliovirus Type 2 (iVDPV2) case in 2015. However, on average, three cases were classified as polio compatible with each year of the study.

**Conclusion:**

South Sudan met the two key surveillance performance indicators and had a sensitive AFP surveillance system during the period studied. We recommend intensifying surveillance activities in the former conflict-affected states and counties to maintain polio-free status.

## Introduction

Acute Flaccid Paralysis (AFP) surveillance backed by laboratory investigation of stool specimens is one of the four strategies that the Global Polio Eradication Initiative uses to measure the progress towards the polio eradication goal [1-3]. The World Health Organization (WHO) Africa region has made quite impressive progress in interrupting wild poliovirus and maintaining a polio-free status since the last case was reported in Nigeria in August 2016 [4]. Nonetheless, outbreaks of circulating vaccine-derived poliovirus type 2 (cVDPV2) had been reported in several countries of the region following the withdrawal of tOPV [5].

South Sudan has been implementing polio eradication activities while being then in Sudan. Since then, South Sudan has registered significant success resulting in the interruption of transmission of wild poliovirus (WPV) in 2009 and wild poliovirus-free status by the African Regional Certification Commission (ARC) in June 2020. Nevertheless, soon after her independence with a peaceful referendum in 2011, a civil war broke out in 2013 and again in 2016, creating ongoing political instability leading to a protracted communal armed conflict accounting for extreme humanitarian crises. This led to a significant destruction of health facilities and social services, creating more challenging circumstances to provide even the basic health service. Parallel to this, surveillance and immunization activities were hampered due to insecurity, displacement, and geographical inaccessibility that left a blind spot in the sensitivity of the surveillance system in some areas. Nonetheless, the surveillance system has overcome many challenges using innovative strategies such as trained community informants, community leaders, and recruiting field staff from the local population to ensure that insecure and hard-to-reach areas are also covered.

South Sudan’s AFP surveillance system is complemented by collecting three stool specimens from close contacts. A study conducted in South Sudan from 2012 to 2016 indicated that 4,687 children contact specimens were collected for 1,637 index AFP cases, and healthy child stool specimens have been collected each month if a county is silent for over 6 months until the county reports a none polio AFP case [6]. Furthermore, a community-based surveillance system was established in the three conflict-affected states following the cVDPV2 outbreak in 2014 to cover the most inaccessible war-affected on top of community informants in all other areas of the country [5]. Moreover, South Sudan´s surveillance system relies on an active surveillance system using Integrated Supportive Supervision (ISS) tool using Open Data Kit (ODK) [7]. The surveillance system also is reviewed by a team of external evaluators every 2-3 years, with the last review done in September 2017 and to further complement the routine AFP surveillance system, environmental surveillance, auto visual AFP case detection, and reporting, reverse cold chain monitoring mechanisms, were introduced [8]. The study aims to evaluate the sensitivity of AFP surveillance in South Sudan using the WHO recommended surveillance standards and highlight the progress and challenges.

## Methods

**Study area:** South Sudan is one of the youngest countries globally, with decades of war and conflict formed in 2011 after a peaceful referendum and ongoing conflict since 2013. The country is bordered to the north by Sudan, to the east by Ethiopia, to the southeast by Kenya, to the South by Uganda, to the southwest by the Democratic Republic of Congo, and the west by the Central African Republic. The population of South Sudan is estimated to be 13.8 million as of December 2019, based on the 2008 census projection. Administratively the country is subdivided into 10 states and 80 counties.

**Study design:** we conducted a retrospective descriptive quantitative study design using the available secondary data reported to the national AFP surveillance database from 2014-2019.

**Study population:** all reported AFP cases that fulfilled the case definition and were verified by trained field officers were included in the study.

**Acute flaccid paralysis surveillance system:** the surveillance system in South Sudan is centered around the health facilities as in many other countries. However, enhanced community-based surveillance was instituted in the three conflict-affected states following the cVDPV2 outbreak in 2014. Furthermore, surveillance networks include government, private, and alternative community healing/treatment facilities that are prioritized into high, medium, and low priority sites. All facilities are mandated to report any suspected cases of AFP and weekly zero reports of AFP. At each administrative level, there are designated Officers who follow the day-to-day operation and conduct active surveillance visits to priority surveillance sites.

**Case definition:** an AFP case is a child < 15 years of age presenting with sudden onset of flaccid paralysis or muscle weakness due to any cause, or any person of any age with paralytic illness if clinicians suspect poliomyelitis.

**Investigation of cases:** an initial investigation of suspected AFP cases conducted by health workers and verified by trained field staff, using a standard case investigation form to capture demographic, clinical, and epidemiological information with 60 days follow up investigation of inadequate cases.

**Laboratory sample collection and testing:** two stool specimens were collected from AFP cases meeting standard case definition with an interval of 24 or 48 hours apart. The collected stool specimens were transported to the national public health laboratory through humanitarian flights with an appropriate reverse cold chain system (2-8°C) and stored at the national level at -20°C until further shipped to Uganda Virus Research Institute (UVRI) for analysis. The condition of the specimens was monitored through tracking mechanisms at each level and recorded in the database. Nearly all specimens are transported to the national level through air flights. Additionally, for each index case, one sample was collected from at least three close contacts irrespective of the geographical areas and stool adequacy.

**Final classification:** all reported inadequate AFP cases were classified by the National Polio Expert Committee (NPEC) supported by the secretariat. All adequate cases are classified automatically using a virological classification scheme.

**Data collection and analysis:** the national AFP database collected through the routine surveillance system and stored at the national level from January 2014 to December-2019 was used. Several indicators measured the sensitivity of the surveillance system with a primary focus on two main indicators: i) non-polio AFP rate 2/100000 children < 15 years; and ii) two adequate stool specimens collected at least 24-48 hours´ apart within 14 days from onset of paralysis and reached laboratory in good condition. For this analysis, an electronic MS Access database was exported into the MS Excel and EPI Info for Windows version 7 (Centers for Disease Control and Prevention, Atlanta, United States) to generate a descriptive analysis, frequencies, tables, and graphs. All surveillance performance indicators were evaluated using the WHO-recommended surveillance standard, and the calculation method followed standard methods.

### Definitions of terms

**Confirmed polio case:** a suspected AFP case with WPV isolation from a stool sample.

**Non-polio AFP cases:** discarded cases are non-polio AFP cases classified by the National Expert Committee after an in-depth review of the cases that exclude all WPV, VDPV, and compatible cases.

**Inadequate cases:** cases detected over 14 days from the date onset of paralysis and arrival of the specimens, at the laboratory in bad condition.

### Main AFP surveillance indicators

**Non-polio acute flaccid paralysis rate:** an indicator of the sensitivity of the surveillance system. As per the guideline the system should detect at least two AFP cases per 100,000 children below the age of 15 years.

**Stool adequacy:** defined as two stool specimens collected from an AFP case 24-48 hours apart and within 14 days of onset of symptoms arriving in the lab in good condition. At least 80% of all AFP cases should have adequate stool specimens

**Ethical approval and consent:** we used the secondary data collected and stored at the national level. Individual verbal consent was received during case investigation and stool sample collection as well as filling of the required information in the AFP case-based form. Administrative clearance for publication of this editorial was provided by the Ministry of Health of South Sudan and WHO (WHO e-Pub IP-00331531-EC) to publish the result. Moreover, the Research Ethics Review Board of Ministry of Health provided clearance for the publication of the manuscript under (MoH/RERB/D.03/2022) clearance number. Moreover, the Research Ethics Review Board of Ministry of Health provided clearance for the publication of the manuscript under (MoH/RERB/D.03/2022) clearance number.

## Results

From 2014 to 2019, a total of 2,212 cases of AFP in children under the age of 15 years were reported nationwide by the surveillance system of South Sudan. An average of 369 AFP cases were reported annually, from all 10 states and all counties. Among the 2,212 cases analyzed, 1,115 (50.6%) were male, and the mean age of the children was 3.2 years with a standard deviation of 2.6 years. The largest proportion (76.4%) involved cases are < 5 years old. However, since 2016 a lower percentage of under-5-year-old has been documented. The majority of 1,724 (80%) of the reported cases were detected within 7 days of the onset of paralysis. However, it decreased from 84.6% in 2014 to 78.5 % in 2019, and most (80.9%) cases were investigated within 48 hours of notification with a decline from 2018 onwards (Table 1).

**Table 1:** characteristics of AFP cases reported 2014-2019, South Sudan

Description							
	2014	2015	2016	2017	2018	2019	Total(%)
Total AFP cases reported	322	331	323	388	448	400	2212
Age <5 years	260(80.7%)	278(84.0%)	250(77.4%)	293(75.5%)	318(71.0%)	291(72.8%)	1690(76.4%)
Age ≥5 years	62(19.3%)	53(16.0%)	73(22.6%)	95(24.5%	130(29.0%)	109(27.2	522(23.6%)
Total	322(100%)	331(100%)	323(100%	388(1100)	448(100%)	400(100%)	2212(100%)
Female	144(45%)	163(49.2%)	157(48.9%)	200(51.8%)	226(50.7%)	199(49.9%)	1089(49.4%)
Male	176(55%)	168(50.8%)	164(51.1%)	186(48.2%)	221(49.3%)	200(50.1%)	1115(50.6%)
Total	320(100%)	331(100%)	321(100%)	386(100%)	447(100%)	399(100%)	2204(100%)
OPV zero dose	10(3.1%)	12(3.6%)	8(2.5%)	10(2.6%)	13(2.9%)	25(6.3%)	78(3.5%)
OPV 1-3 doses	24(7.6%)	29(8.8%)	20(6.2%)	42(10.8%)	36(8.0%)	47(11.8%)	198(9.0%)
OPV 4+ doses	288(89.4%)	290(87.6%)	295(91.3%)	336(86.6%)	399)89.1%)	328(82.0%)	1936(87.5)
Total	322(100%)	331(100%)	323(100%)	388(100%)	448(100%)	400(100%)	2212(100%)
% of cases with fever at onset	319(99%)	326(98.5%)	319(98.8%)	379(98.4%)	436(97.5%)	393(98.3%)	2172(98.4%)
% of cases paralysis progressed within 3 days	295(91.6%)	306(94.5%)	308(95.4%)	354(91.5%)	402(90.5%)	363(91.0%	2028(91.9%)
% of cases with asymmetrical paralysis	255(79.4%)	293(89.3%)	292(91.3%)	288(80.5%)	300(75%)	254(64.1%)	1682(79.2%)
% of cases investigated within 48 hours of notification	276(85.7%)	289(87.3%)	267(82.7%)	313(80.7%)	348(77.7%)	296(74.0%)	1789(80.9%)
% cases detected within 7 days	264(84.6%)	280(86.2%)	270(85.2%)	293(77.7%)	311(70.4%)	307(78.5%	1725(80.0%)

AFP: acute flaccid paralysis; OPV: oral polio vaccine; %: percentage

The clinical characteristics of the reported AFP cases indicated that the great majority (98.4%) had a fever at the onset of the paralysis, while in 2028 (91.9%) of these cases, had complete flaccid paralysis within 3 days of the onset of paralysis. Our study also showed that 1,936 (87.5) of the AFP cases reported during the study period had received 4+ doses of OPV, while 3.5% of the children was zero or unknown OPV doses. Over the study period, the proportion of cases that received 4+ doses remained above 82%. The arrival of two adequate stool specimens collected in 24-48 hours at the laboratory in “good condition” was consistently above the established standard of 90% throughout the study period. The Non-Polio Enterovirus (NPENT) isolation rate remained well above the certification level (10%) during the study period, except for 2014. On the other hand, the average days of transporting the specimen from the field to the national level are 14 days and ranged from 12 days in 2014 to 16 days in 2018, and the number of silent counties gradually decreased from 15 in 2014 to 3 in 2019. On the other hand, at least three close contact specimens were collected in an average of 96.4% of the index cases (Table 2)

**Table 2:** surveillance performance indicators final classification of cases, 2014-2019, South Sudan

	2014	2015	2016	2017	2018	2019	Average/total
% of cases with at least three contact specimens collected	297	322	312	363	440	387	297
	(96.40%)	(96.40%)	(96.40%)	(96.40%)	(96.40%)	(96.40%)	(96.40%)
% of stool samples arriving at a national lab in good condition	307	328	318	374(96.4%)	447	399	300
	(96.50%)	(99.10%)	(98.50%)		(00%)	(99.80%)	(82%)
% of stool specimens from which non-polio enterovirus isolated	9.7	11.2	13.2	10.8	7.6	13.8	10.9
% of stool specimens from which sabin like isolates	2.2	4.5	3.2	4.4	3.5	2.5	3.38
% of counties achieved two main indicators	58	71	66	47	57	68	61
% of counties achieved NP_AFP rate	68	77	80	67	84	89	78
% of counties achieved stool adequacy	68	77	71	52	52	73	66
# of silent counties	15	10	4	15	7	3	9
Average days sample arrival from field to national level	12	14	13	14	16	15	14
Timelines of zero AFP reporting	73	86	84	85	83	85	82.7
Inadequate cases reviewed by NPEC			25	50	70	42	0
Inadequate cases discarded			25	45	62	36	0
**Final classification**							
WPV cases detected	0	0	0	0	0	0	0
cVDPV2 cases detected	2	0	0	0	0	0	2
Discarded cases	318	329	323	440	392	399	2201
Compatible cases	2	1	0	5	8	6	24

NP_AFP: non-polio acute flaccid paralysis; NPEC: National Polio Expert Committee; WPV: wild poliovirus; cVDPV2: circulating vaccine-derived poliovirus type 2

**Figure 1:**
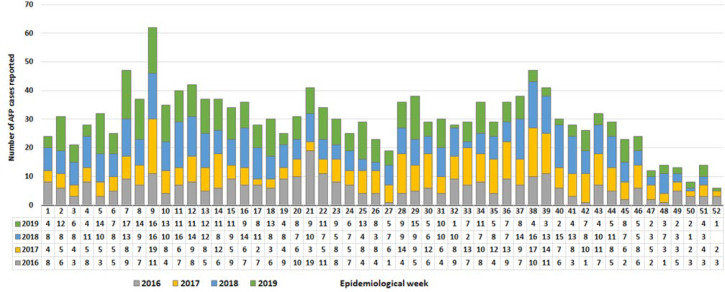
acute flaccid paralysis cases reported by week of onset 2014-2019, South Sudan

**Figure 2:**
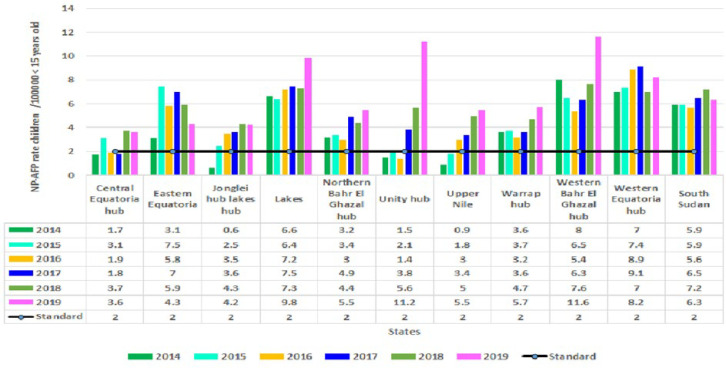
non-polio acute flaccid paralysis rate by states and year, 2014-2019, South Sudan

The analysis results showed that an average of 47 cases were detected per week. However, a significant increase in the number of detected cases was observed from weeks 7 to 14 and again from weeks 34 to 40 of each year. The number of cases started to gradually decrease each year since week 41 as the year’s end (Figure 1). The country consistently achieved the NP-AFP rate during the entire study period, well above the standard (2/100,000 children < 15 years). The non-polio AFP rate averaged 6.2/100,000 children < 15 years old, while it increased from 5.9/100,000 < 15 years old in 2014 to 6.3 in 2019. Six states had consistently achieved a non-polio AFP rate well above the target of 2/100,000 children < 15 years of age during the entire study period, while six, eight and 9 states had achieved in 2014, 2015 and 2019 respectively. In the last 3 years of the study period, all states had persistently achieved the target except Central Equatoria state. The analysis of the NP-AFP rate at the county level showed that an average of 78% of counties met the target, an increase from 68% in 2014 to 89% in 2019 (Figure 2).

**Figure 3:**
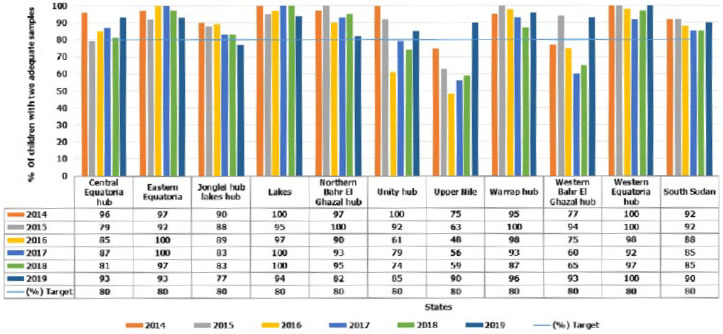
percent of children with two adequate samples by states and year, 2014-2019, South Sudan

The stool adequacy was persistently above the target of 80% throughout the study period. Though certification level was maintained, there was a decline in the proportion of stool adequacy in 2017 and 2018. The proportion of two adequate stool specimens at the state level varied, with only five of the ten states persistently attaining the minimum required standard over the study period. There was an insistently low performance in Unity, Western and Upper Nile Bahr El Ghazal states for 3, 4 and 5 years respectively. Upper Nile was persistently low performing for 5 years except in 2019. Central Equatoria state fell below the stool adequacy standard in 2015, while Jonglei state fell below the target in 2019. Nevertheless, all states had attained stool adequacy in 2019 except Jonglei state which fell below the standard. The performance at the county level indicates, overall, 61% of the counties attained this indicator with a gradual increase from 68% in 2014 to 73% in 2019 (Figure 3).

**Figure 4:**
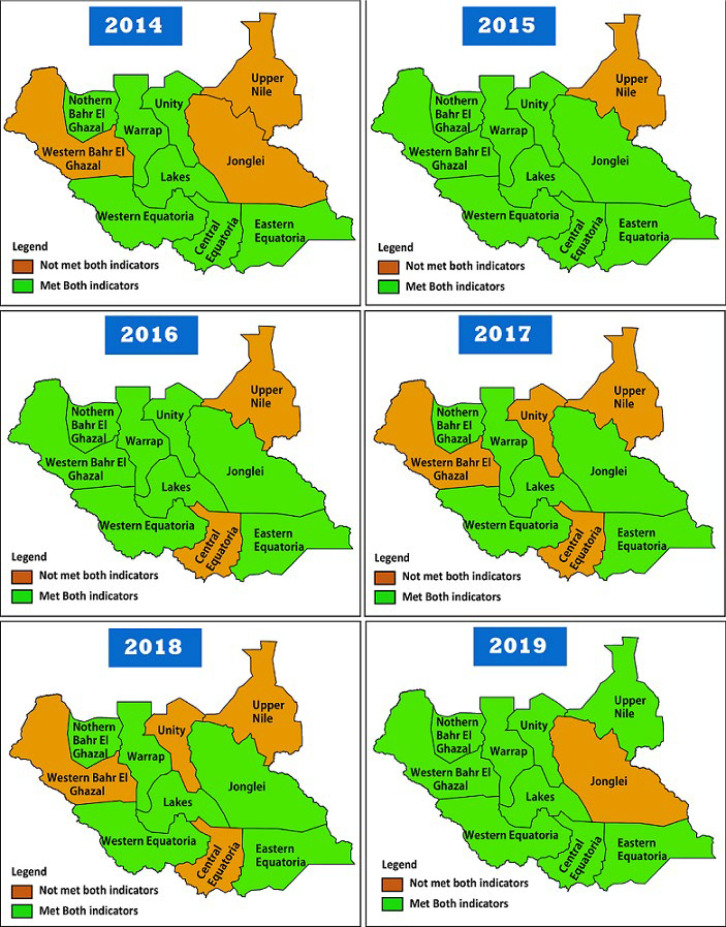
combined two main surveillance indicators (non-polio acute flaccid paralysis rate and stool adequacy), 2014-219, South Sudan

The country met the two core indicators in the last six years of the study period. At the state level, however, only five states consistently achieved both indicators over the study period. By 2019, however, all states had managed to meet both core AFP surveillance indicators except for Jonglei state, which fell below the standard for stool adequacy of less than 80%. It is of note that Unity and Western Bahr Ghazal persistently fell below to meet both AFP surveillance core indicators except in 2015 and 2019, while Upper Nile met the two indicators only in 2019 (Figure 4). Further analysis indicated that 61% of the counties met the two main AFP surveillance performance indicators over the study period. A progressive achievement from 58.6% in 2014 to 68% in 2019 (Table 2).

**Figure 5:**
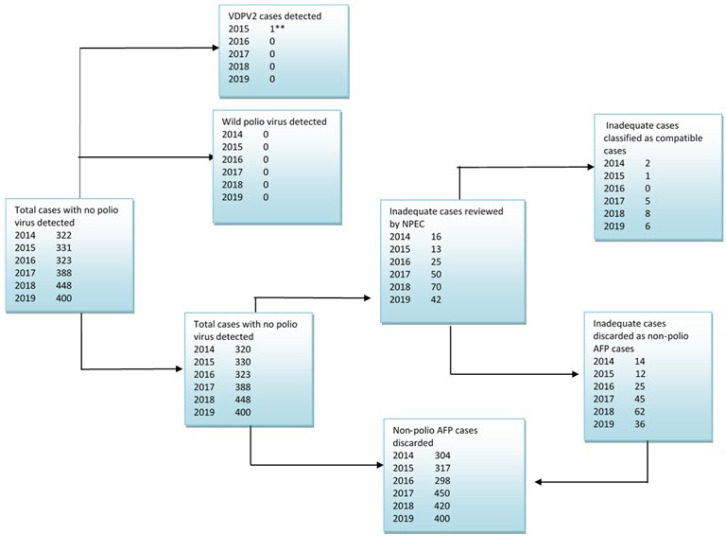
virological classification of acute flaccid paralysis cases 2014-2019, South Sudan

This study indicated that from 2014 to 2019, no WPV case was detected from AFP or environmental samples except that two circulating Vaccine Derived Polio Virus Type 2 (cVDPV2) and one Immunodeficiency Vaccine Derived Polio Virus type 2 (iVDPV2) cases were detected in AFP cases in 2014 and 2015 respectively. Though no WPV case was detected throughout the study period, attenuated Sabin like virus was isolated on average in 3.4% of the stool specimens tested, and over the study period, 22 cases were classified as polio compatible during the study period (Figure 5).

## Discussion

The present study reports over a six-year (2014-2019) period in South Sudan to measure the sensitivity of the AFP surveillance system against WHO recommended standard. Our study demonstrated that South Sudan achieved a consistently high NP-AFP rate throughout the study period exceeding well above the WHO recommended minimum surveillance standard [7]. This agrees with previous studies carried out in Egypt and Nigeria [8,9], but higher than the studies conducted in Ghana, Ethiopia, Kenya, Bangladesh and Nigeria [10-14]. The high non-polio AFP rate at the national level can be attributed to the implementation of various strategies to reach insecure and difficult-to-reach areas through the engagement of local personnel. It may also be facilitated by setting up the community-based surveillance system in some states and engaging community informants including introducing AVADAR, Open Data Kit (ODK) and monitoring of staff accountability [15-18]. However, in the three formerly conflict-affected states, despite all these, there was persistent low stool adequacy over the study period, though all states met both indicators in 2019 except Jonglei. The persistently low stool adequacy may be due to the ongoing interclan conflict or a result of the conflict, which has hampered early case detection and notification of cases, including difficulties in transporting specimens due to a dysfunctional public transportation system.

Overall, despite the conflict, uncertainty and humanitarian crises, we found that South Sudan met the two key surveillance performance indicators throughout the study period and was well above the WHO surveillance standard, and also higher than the studies conducted in Kenya and Bangladesh, Zimbabwe and DRC [7, 12, 13, ,18,19,]. Nevertheless, all states did not meet the two main surveillance indicators except in 2019 where all had achieved both indicators except Jonglei. Similarly, the percentage of specimens that arrived in the laboratory in good condition was also well above the certification standard. However, the delays in specimen transport were extremely exceedingly above the maximum WHO-recommended surveillance standard of three days. Interestingly, despite the harsh transportation and delayed arrival of specimens, we found that the isolation rate of non-polio enteroviruses rate is higher than that most of the studies conducted [18-20]. Similarly, early detection of cases within 7 days of onset of paralysis and case investigation within 48 hours of notification is in line with WHO-recommended standard, but lower than the study conducted in Egypt and Ethiopia [7,8,11]. The low rate of AFP stool specimens arriving in the laboratory within three days of the collection is attributed to the logistic problems of shipping specimens. Except for the Central Equatorial State, all collected specimens from all corners of the country are shipped to the national level by humanitarian flights, and these flights are scheduled weekly. Furthermore, the public transportation system connecting the counties to airstrips are in most cases are not functional.

This study revealed the vaccination status of children with more than four doses of OPV was higher than the study done in Uganda and the national routine vaccination coverage [21,22]. However, it was in agreement with other results found in Ethiopia, Kenya and South Africa, but lower than compared to other studies carried out [8,11- 24]. The high vaccination coverage may be related to repeated supplementary immunization campaigns. South Sudan had conducted 3-4 rounds of supplemental immunization activities each year during the study period. However, high population immunity is mainly for poliovirus types 1 and 3 following the global withdrawal of trivalent oral poliovirus in 2016 and low IPV coverage [25,26]. Consequently, population immunity to poliovirus type 2 continues to decline, and hence the risk of cVDPV2 outbreaks remains high. There was no wild poliovirus isolated from AFP or environmental samples during the study period, except for VDPV2 cases in 2014 and 2015.

The high proportion among children aged < 5 years can be attributed to the low standard of living associated with hygiene and sanitation, which leads to infections. Our study also noted that the highest proportion of AFP cases were reported from three states (i.e., Warrap, Lakes, and Western Equatoria). This can be explained by the sensitive AFP surveillance system in these states rather than their population size, as these states contribute only 27% of the country’s total population. On the other hand, the high reporting rate in Warrap may have been facilitated by an AVADAR surveillance system in Gogrial West County since June 2018, as most of the cases were reported through the AVADAR system.

We identified some limitations in our study that could adversely affect our study. First, the sub-optimal data with missing and incomplete variables at the begging of the program, however, the data set was cleaned, and some missing variables were captured from the field and case-based forms stored at the national level before data analysis. On the other hand, those grossly incomplete datasets were excluded from the analysis. Though there are limitations, our study highlighted the major strengths and weaknesses of the surveillance system that can assist decision-makers

## Conclusion

We concluded that the AFP surveillance system in South Sudan was effective in meeting the WHO recommended surveillance standard for the last six years, and it is highly sensitive enough to detect minimal circulation due to importation or re-emergence of poliovirus in the country. However, sub-optimal surveillance does exist in the former conflict-affected states and Western Bahr Ghazal state, mainly with low stool adequacy, which needs to be improved. To maintain polio-free status, we recommend maintaining and strengthening ongoing surveillance activities, with a particular emphasis on conflict-affected states and counties. We also suggest re-designing strategies to fast-track stool transportation mechanisms to cut down the delay to meet the WHO standard by looking at other options for transporting specimens and monitoring using Log Tag in counties with the critical concern of delayed shipping of stool specimens. Taking into consideration the contribution of United Nation Humanitarian air service flight in specimen shipment, we recommend a frequent detailed discussion with management of humanitarian flight to reduce delays of shipment. Furthermore, we recommend ensuring good quality of data, harmonization of data with laboratory and institute community-based surveillance to early detect cases, enhanced monitoring, and analysis of surveillance data completeness.

### What is known about this topic

- A sensitive and effective surveillance system is critical to eradicating poliomyelitis;
- Active surveillance for AFP has been designed for AFP surveillance to rule out undetected circulation;
- It is also critical to achieving main surveillance indicators as well as to sensitize and build the capacity of health workers at the peripheral level.

### What this study adds

- Security challenges played a significant role in achieving surveillance indicators in remote and inaccessible areas. However, in this case, using community leaders and informants greatly improved the sensitivity of the surveillance system;
- For the first time, the AFP surveillance data in South Sudan has been systematically analyzed, providing researchers with an opportunity to undertake further detailed research on various factors that affect the sensitivity of the surveillance system in high-risk countries;
- There is a significant improvement in the AFP surveillance indicators of the country, and the surveillance is sensitive enough to pick up any poliovirus circulating. However, there is a need to continue the current momentum to maintain surveillance sensitivity.
